# [Corrigendum] Coenzyme Q10 alleviates sevoflurane-induced neuroinflammation by regulating the levels of apolipoprotein E and phosphorylated tau protein in mouse hippocampal neurons 

**DOI:** 10.3892/mmr.2026.13995

**Published:** 2026-08-20

**Authors:** Man Yang, Naqi Lian, Yang Yu, Yaoqi Wang, Keliang Xie, Yonghao Yu

Mol Med Rep 22: 445–453, 2020; DOI: 10.3892/mmr.2020.11131

Following the publication of the above article, a concerned author drew to the authors’ attention that they had apparently used an inappropriate antibody in their study: In the Materials and methods section, the authors had reported the use of Abcam's recombinant antibody against plant homeodomain (PHD) finger protein 1 (a.k.a. PHF1; cat. no. ab184951) to probe for the unrelated protein, tau paired helical filaments (PHFs). The authors acknowledged that they had made a methodological error: They were intending to detect tau phosphorylation at Ser396/404, but mistakenly had used the antibody against PHD finger protein 1 (cat. no. ab184951; Abcam); as a result, the western blot results shown in Figs. 3 and 7 for protein PHF1 were invalid. This error went unnoticed since the two antibodies shared the same abbreviation (PHF1), and the banding patterns obtained with the incorrect antibody appeared similar in trend to what the authors had expected to have found, which unfortunately masked the mistake during their internal data review. The Editor instructed the authors to repeat the PHF1 western blot experiments using the correct PHF1 antibody (Tau-pSer396/404; cat. no. 87061-1-RR; Proteintech Group, Inc.). The new experiments yielded reproducible and valid results, and the authors have replaced the original data relating to protein PHF1 in Figs. 3 and 7; the revised versions of these figures are shown on the next page.

Note that these errors did not affect the results or the main conclusions reported in the study. All the authors approve of the publication of this corrigendum, and are grateful to the Editor of *Molecular Medicine Reports* for allowing them the opportunity to publish this. The authors regret their oversight in selecting the wrong antibody for these specific western blot experiments, and apologize to the readership for any inconvenience caused.

## Figures and Tables

**Figure 3.**
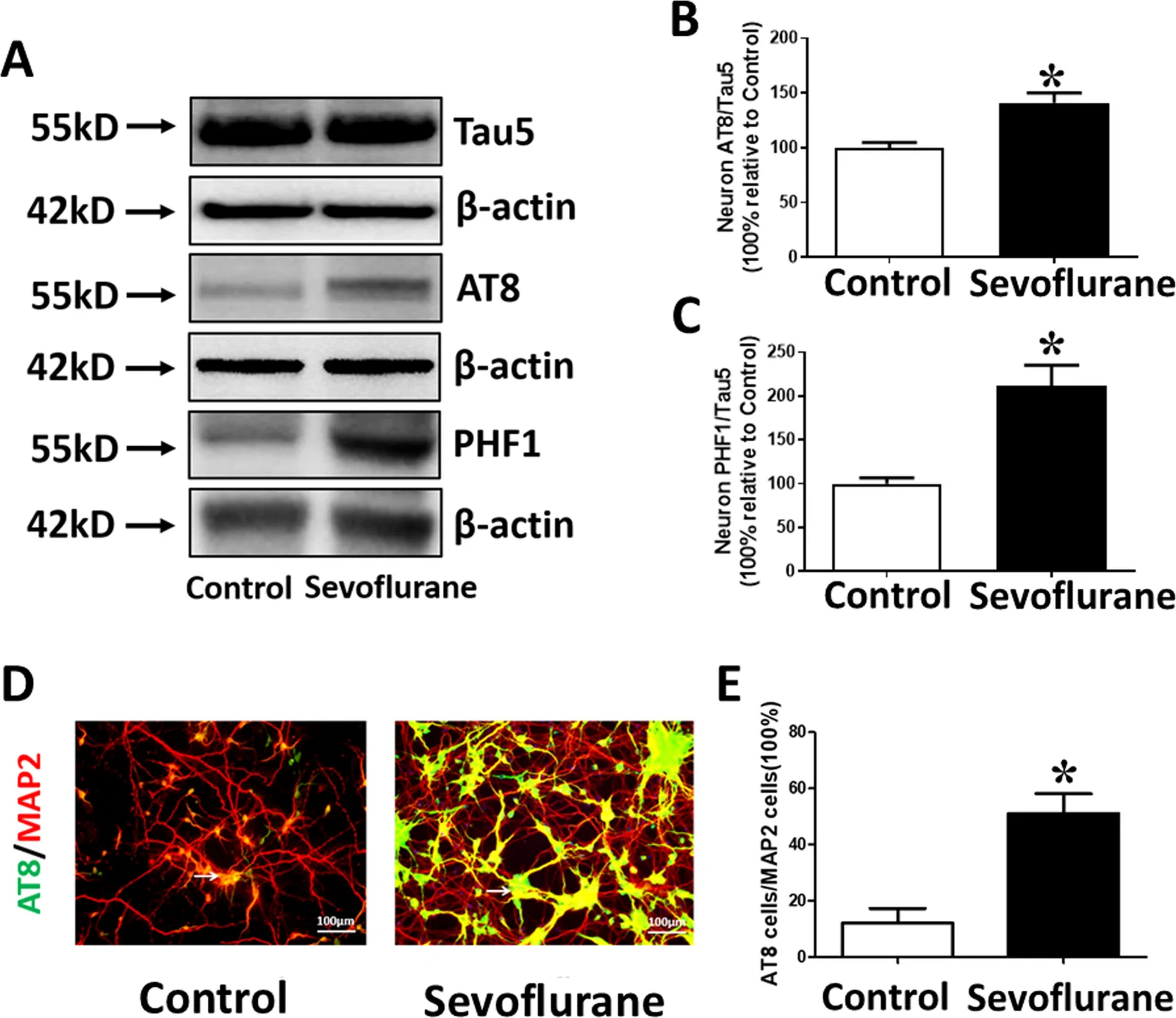
Effects of sevoflurane anesthesia on tau phosphorylation in mouse hippocampal neurons. (A) Western blotting was used to compare the expression levels of Tau5, AT8 and PHF1 in neurons between the control and sevoflurane groups. (B and C) The western blotting results of AT8 and PHF1 expression were represented as the ratios of (B) AT8 to Tau5 and (C) PHF1 to Tau5. (D) Immunofluorescence was used to test the expression of AT8 in neurons of both groups. (E) AT8 expression in immunofluorescence staining was calculated as the ratio of AT8-positive neurons (green) to MAP2-positive neurons (red) in the stained area. n=6 in western blotting and n=3 in immunofluorescence per group. *P<0.05. Tau5, total tau; AT8, Tau-PS202/PT205; PHF1, Tau-PSer396/404; MAP2, recombinant microtubule associated protein 2.

**Figure 7.**
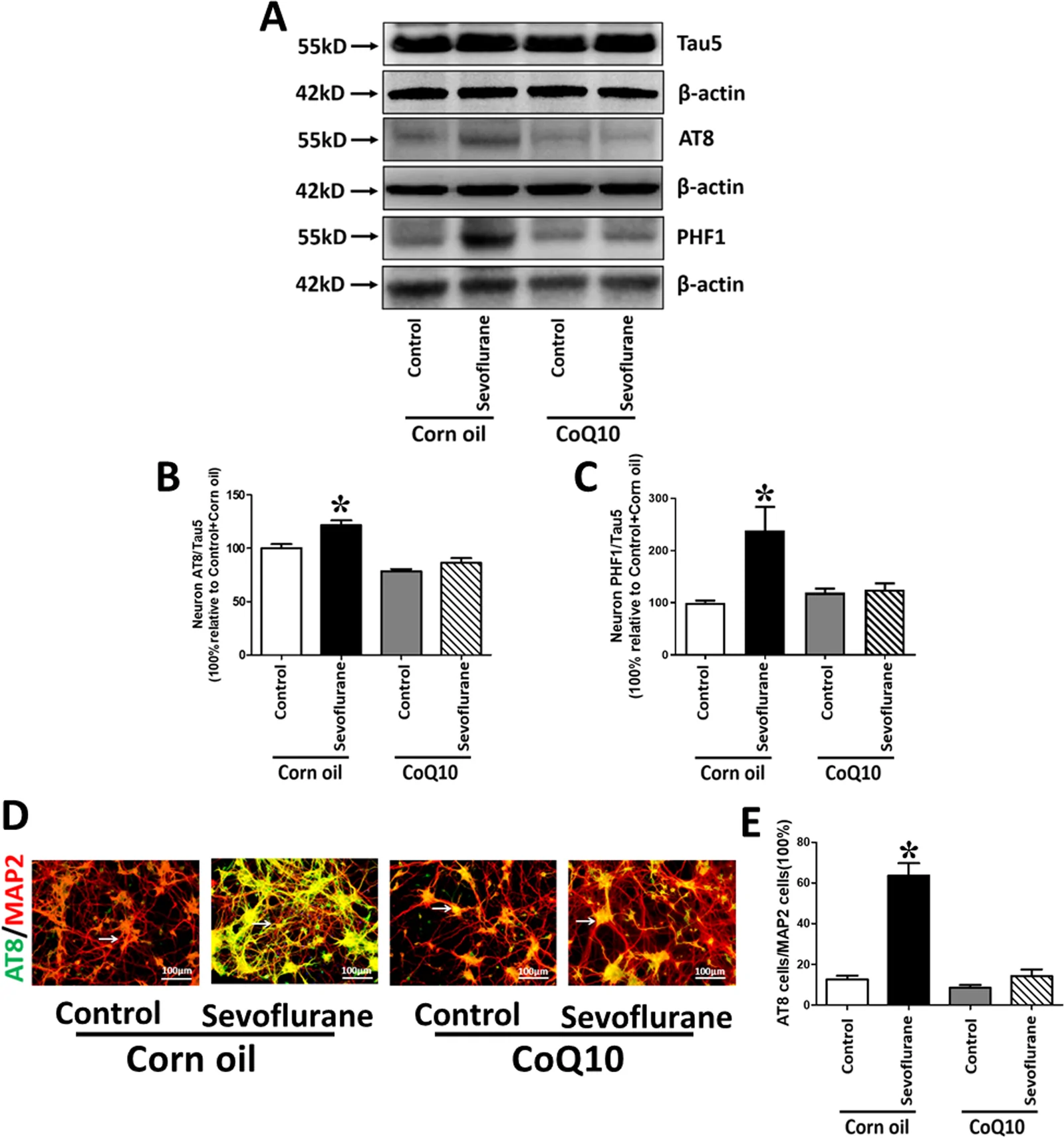
Effects of CoQ10 on tau phosphorylation in mouse hippocampal neurons following sevoflurane anesthesia. (A) Western blotting was used to measure the expression levels of Tau5, AT8 and PHF1 in neurons. (B and C) The western blotting results of AT8 and PHF1 expression were presented as the ratios of (B) AT8 to Tau5 and (C) PHF1 to Tau5. (D) Immunofluorescence assay was used to evaluate the expression of AT8 in the neurons of all the groups. (E) AT8 expression in immunofluorescence staining was calculated as the ratio of AT8-positive neurons (green) to MAP2-positive neurons (red) in the stained area. n=6 in western blotting and n=3 in immunofluorescence per group). *P<0.05 vs. control+corn oil. CoQ10, coenzyme Q10; Tau5, total tau; AT8, Tau-PS202/PT205; PHF1, Tau-PSer396/404; MAP2, recombinant microtubule associated protein 2.

